# The efficiency of the medical role within a Single Point of Access (SPA) Service in reducing the number of clinic appointments required

**DOI:** 10.1192/bjo.2021.824

**Published:** 2021-06-18

**Authors:** Anca Bradu, Sam Nayrouz

**Affiliations:** West London NHS Trust

## Abstract

**Aims:**

The SPA service takes referrals from general practitioners (GPs), medical professionals, the London Ambulance service, the London Police, psychology and social services, and from patients themselves and their family members or support groups. Some of these referrals require input from secondary care, but others can be solved within primary care if given specialist advice, this minimizing the time spent by patients in the healthcare system and minimizing also the NHS costs.

Our aim was to evaluate the implementation of the Advice from Medics Service in a 1-year period.

**Method:**

We examined a random sample of 200 referrals between 1st of April 2019 and 31st of March 2020 out of all referrals that were considered, after the triage, to be appropriate for an advice on treatment provided by the medics as an alternative to a clinic appointment in secondary care. We collected information from the electronic patient records regarding the dates of referrals, the senders of referrals, the type of referrals, the age and gender of patients and the reasons to be referred, and finally we analysed the outcome of the referrals and compared it with the action requested.

**Result:**

Of the 200 referrals, 113 were for female patients and 87 for male patients. The age of patients was between 18 and 91 years old, with a median of 43 years old.

The person/authority making the referral was the GP in 179 cases, and others in 21 cases.

The referrers asked for review in 74 cases, urgent review in 2 cases, review and advice in 31 cases, only advice in 46 cases, and did not state the type of referral in 47 cases.

The primary pathology implied was affective in most of the cases (122), followed by psychotic (31) and neurotic (22), organic (8), of personality (5), hyperkinetic disorders (5), due to substance misuse (4), of psychological development (2) and learning disability (1).

The outcome of the referrals was as follows: 19 patients (9.5%) were seen by the Crisis Team, 11 (5.5%) were referred to other teams, 4 (2%) did not engage with SPA, and the rest of 166 (83%) referrals were solved with advice.

**Conclusion:**

The outcome was extremely favourable as the majority of referrals requested medical review but most of them (83%) were solved with specialist advice to GPs or other professionals, highlighting that the implementation of the Advice from Medics Service has been an improvement to the SPA.

